# Conformity to the descriptive norms of people with opposing political or social beliefs

**DOI:** 10.1371/journal.pone.0219464

**Published:** 2019-07-10

**Authors:** Campbell Pryor, Amy Perfors, Piers D. L. Howe

**Affiliations:** School of Psychological Sciences, University of Melbourne, Victoria, Australia; Middlesex University, UNITED KINGDOM

## Abstract

The descriptive norm effect refers to findings that individuals will tend to prefer behaving certain ways when they know that other people behave similarly. An open question is whether individuals will still conform to other people’s behaviour when they do not identify with these other people, such as a Democrat being biased towards following a popular behaviour amongst Republicans. Self-categorization theory makes the intuitive prediction that people will actively *avoid* conforming to the norms of groups with which they do not identify. We tested this by informing participants that a particular action was more popular amongst people they identified with and additionally informed some participants that this action was unpopular amongst people they did not identify with. Specifically, we presented descriptive norms of people who supported different political parties or had opposing stances on important social issues. Counter to self-categorization theory’s prediction, we found that informing participants that an action was unpopular amongst people they did not identify with led participants’ preferences to shift away from that action. These results suggest that a general desire to conform with others may outpower the common ingroup vs outgroup mentality.

## Introduction

Our choices and judgements are influenced by the choices and judgements of other people [1, 2]. In particular, we tend to prefer to behave in certain ways when we know that most other people also behave that way. This descriptive norm effect has been successfully used to encourage various prosocial behaviours such as decreasing tax evasion [3], decreasing energy use [4] and increasing organ donor registrations [5], though they can also encourage anti-social behaviour such as corruption [6, 7], even in the presence of strong moral pressures against the behaviour [8]. Given the importance that descriptive norms play in our decisions, it is both theoretically and practically beneficial to understand why and when we conform to descriptive norms.

Self-categorization theory is a prominent explanation of both the descriptive norm effect [9], and a range of other social phenomena such as ingroup favouritism [10], stereotyping [11] and power dynamics [12]. Self-categorization theory proposes that an individual’s identity is linked to the social groups with which they identify (their ‘ingroups’) [13]. In an effort to maintain a personal sense of ingroup identity, self-categorization theory proposes that people will adopt the characteristics of these salient social ingroups, leading to ingroup norm conformity.

Through this emphasis on group identification, self-categorization theory predicts that the degree to which an individual will conform with the norms of an ingroup will be determined by how strongly they perceive themselves to be a member of that ingroup [9]. Consistent with this prediction, Wellen, Hogg and Terry [14] found that people were more likely to conform to the norms of a particular group when the salience of their membership with that group was experimentally heightened by having them describe how they were similar to other members of the group. Similarly, other studies have also found that the more a participant identifies with the ingroup, the more strongly they are influenced by that group’s norms [15, 16]. However, Rimal & Real [17, 18] failed to replicate this result, suggesting group identity may not always determine whether individuals adhere to the ingroup norm, and self-categorization theory’s account of the norm effect may be incomplete. This underscores the importance of testing other predictions of self-categorization theory with respect to the norm effect.

A key prediction of self-categorization theory is that people will actively avoid conforming to behaviour that is popular or endorsed by groups with which they do not identify (outgroups) [9]. Just as it predicts that people will conform to salient ingroup norms in order to maintain their sense of ingroup identity, self-categorization theory predicts that people will avoid conforming to salient outgroup norms for a similar reason; remaining distinct from the outgroup helps people to maintain their sense of ingroup identity. It follows that individuals should conform even more strongly to an ingroup norm when an outgroup tends to behave in the opposite way.

In contrast, explanations of the descriptive norm effect that do not focus on self-categorization theory typically ignore group identity and make the more general assumption that people will follow “what most people do” [19]. This alternative hypothesis predicts that people will conform to the overall norm, responding similarly to ingroup and outgroup norms.

To our knowledge, no previous study has looked at whether ingroup and outgroup descriptive norms have different effects on the behaviours people prefer to conform to. However, previous studies have looked at the effects of ingroups vs outgroups for other social phenomena. The concepts of ingroups and outgroups was popularized by self-categorization research into the minimal group paradigm, where it was found that people are biased towards helping an ingroup member over an outgroup member [20], even when group membership was anonymous and designated based on trivial criteria, such as preference for a particular painting.

Relevant to our current study, Hogg, Turner and Davidson [9] informed their participants that a small set of four other participants from an outgroup (as defined by having different attitudes to the current participant) supposedly favoured a risky (cautious) option. They found that this led the current participant to predict that their own ingroup would favour more cautious (risky) options. A similar study by Krizan and Baron [21] failed to replicate this effect. In particular, they did not find that presenting information that a small outgroup was cautious had a significant effect on the decisions of their participants. However, this could be attributed to the fact that the outgroup information was presented briefly amidst 20 minutes of ingroup discussion. Cruwys et al. [22] found that when participants saw someone from their own university eat a lot of popcorn, they tended to eat more popcorn too and vice versa when they saw the ingroup member eat very little popcorn. This effect slightly reversed when seeing a single outgroup member eat a lot or very little popcorn (as predicted by self-categorization theory), though this effect was not significant. The extent to which results from these studies would generalise to descriptive norms is unclear, given small or single-person outgroups provide little information about what behaviour is typical of the broader outgroup.

### Aim and hypotheses

The aim of this study was to test the prediction of self-categorization theory that people’s behaviour will shift away from behaviour common amongst an outgroup, against the alternative hypothesis that people will simply conform to the overall descriptive norm. We tested this by presenting participants with an ingroup descriptive norm favouring a certain option and additionally presenting half of the participants with an outgroup descriptive norm that favoured the alternative option. Self-categorization theory predicts that, in an effort to remain distinct from the outgroup, participants will conform more to the ingroup descriptive norm when an opposite outgroup descriptive norm is shown. The alternative hypothesis is that people will conform to the overall descriptive norm, such that conformity with the ingroup descriptive norm will decrease when an opposite outgroup descriptive norm is presented.

It is worth noting that in the current paper we do not create and designate participants to ingroups and outgroups but instead draw their attention to pre-existing social categories, specifically based on political partisanship and attitude towards social issues such as feminism, gun control and religion. Groups based on these political and social attitudes are constantly in opposition within the US and play an important role in people’s social identity [23].

To pre-empt our results, we found that participants’ preferences shifted towards (rather than away from) the behaviour that was popular amongst the outgroup descriptive norm, when it was presented. This occurred even when defining the ingroup and outgroup based on political identity or social issues that participants indicated that they cared about, such as gun control. These results are inconsistent with self-categorization theory and instead argue for a more general mechanism whereby people tend to conform to both the outgroup and ingroup descriptive norm.

## Experiment 1

### Method

#### Participants

301 participants (M_age_ = 40 years, 60% female) from the United States of America were recruited via Mechanical Turk and paid US$0.65 for participation. The experiment took around 3 minutes to complete and was completed in a web browser. Informed consent was obtained in all experiments reported here. Ethics approval for all experiments reported here was granted by the University of Melbourne Human Research Ethics Committee. All experiments were carried out in accordance with the relevant guidelines and all participants gave informed written consent. Data collection and analysis were not performed blind to the conditions of the experiments.

#### Procedure

After providing basic demographic information about age and sex, participants were asked to select which out of a set of nine topical social issues, such as gun control and immigration, they cared most about. After selecting the issues they cared about most, participants were presented with a statement about their chosen issue (the full list of these statements is contained in S3 Text). For example, if a participant selected gun control as the issue they cared about most then they were presented with the following statement “Adults should have the right to carry a concealed handgun”. Participants were asked to report the extent to which they agreed or disagreed with the statement on an 11-point Likert scale ranging from -5 (Strongly Disagree) to +5 (Strongly Agree). Their rating of this chosen social issue was then used to define the ingroup and outgroup when subsequently presenting descriptive norms, as outlined below.

Participants were then presented with instructions for the current study. They were told that this study was following on from a previous study that investigated how people feel during a moral dilemma. This background story was included simply to justify the source of the descriptive norms that were later presented. Participants were told that they would be presented with a scenario describing a moral dilemma and have to choose which action they would take and then rate how they would feel about it.

After reading these instructions, participants were presented with the following moral dilemma:

“Imagine you have witnessed a man rob a bank. However, you then saw him do something unexpected with the money. He donated it all to a run-down orphanage that would benefit greatly from the money. You must decide whether to call the police and report the robber or do nothing and leave the robber alone.”

Below this moral dilemma, participants were presented with an ingroup descriptive norm informing them that 60% of previous participants who had agreed with them about their chosen social issue (i.e. members of their political ingroup) chose to act a certain way. Half of the participants were told that their ingroup members mostly chose to “call the police and report the robber” while the remaining half were told that their ingroup members mostly chose to “do nothing and leave the robber alone”. So, for example, if participant X indicated that they cared most about gun control, they might have been told that “approximately 60% of participants who agreed with you about gun restrictions chose to call the police and report the robber”.

Additionally, half of the participants were also that that, in the previous study, 85% of participants that disagreed with them on that issue chose the other option. From the example above, participant X would have been informed that “approximately 85% of participants who disagreed with you about gun restriction chose to do nothing and leave the robber alone”. Thus, our study had a 2 x 2 balanced design: half our participants were told that the ingroup norm favoured one action whereas the remaining participants were told that it favoured the other action; half our participants were presented only with the ingroup norm, whereas the remaining participants were presented with both the ingroup and outgroup norms. For an example transcript, please see S1 Text.

Participants then indicated how they would respond to the moral dilemma on a 6-point Likert scale ranging from “Definitely call the police and report the robber” to “Definitely do nothing and leave the robber alone”. To fit with the backstory presented in the instructions, participants were also asked to rate how good or bad they felt about their chosen action, although these responses were not analysed.

In order to ensure that participants were paying attention, as is especially recommended for Mechanical Turk studies [24], we included an understanding check asking participants which of the following options was true about the previous study described in the instructions:

1. *Participants chose which action they preferred* (correct)
2. *Due to a computer error*, *participants were not allocated equally to imagine performing the different actions* (incorrect)
3. *No data was saved during the experiment*. (incorrect)
4. *The participants completed the experiment with their eyes closed*. (incorrect)

Finally, Postmes, Haslam and Jans’ [25] single-item social identification measure was included after the understanding check to test whether individuals identified with the relevant ingroup and did not identify with the relevant outgroup. This measure simply involves asking participants the extent to which they agree with two statements about whether they identified with the designated ingroup and outgroup. The statements were “I identify with [ingroup]” and “I identify with [outgroup]”, where [ingroup] and [outgroup] were replaced with the appropriate descriptions (e.g. “Pro-Gun Enthusiasts” and “Anti-Gun Advocates”).

#### Design

A 2 (ingroup descriptive norm) x 2 (both norms shown) between-subjects design was used. The independent variable both norms shown refers to whether only an ingroup descriptive norm was shown (both norms shown = 0) or both an ingroup descriptive norm and an outgroup descriptive norm were shown (both norms shown = 1). The variable ingroup descriptive norm refers to whether the ingroup descriptive norm favoured reporting the robber (ingroup descriptive norm = −1) or leaving the robber alone (ingroup descriptive norm = 1). When both the ingroup and outgroup descriptive norms were shown, we randomly varied their ordering. This was done only to control for potential order effects and so was ignored when analysing the data. The dependent variable was participants’ responses on the Likert scale rating the certainty with which they would act a certain way. Any participants that failed the understanding check were excluded because this indicated that they had not paid attention throughout the task. Additionally, any participants that reported being neutral about their chosen social issue was excluded because this prevented us from determining an ingroup and outgroup.

### Results

**Fig 1:**
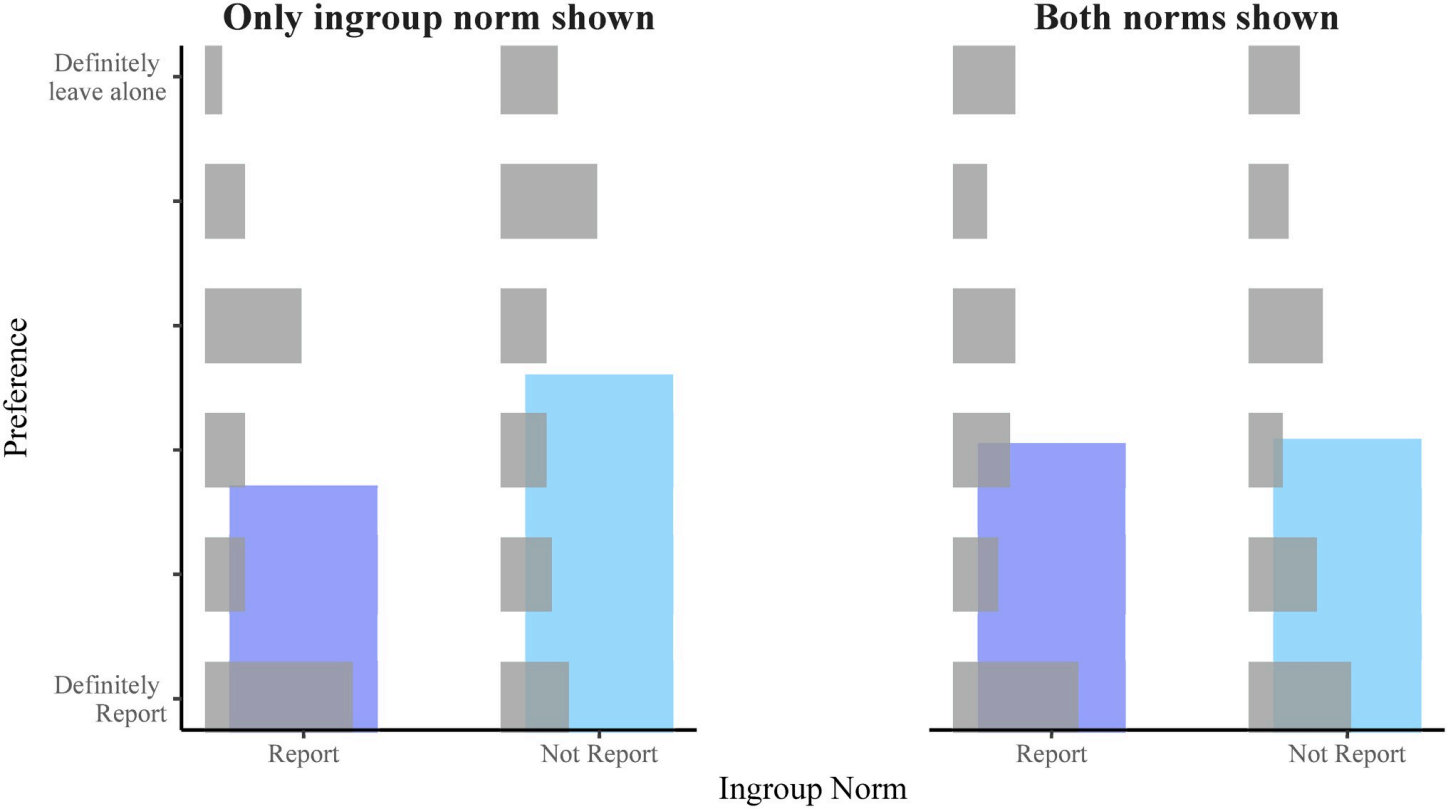
Superimposed bar chart representing responses to the moral dilemma in each condition of Experiment 1. The horizontal grey bars represent the relative proportion of each response in each condition. The vertical blue bars represent the mean response in each condition in order to give a better sense of how the pattern of responses changed in each condition. The results of Experiment 1 are consistent with the alternative hypothesis that people’s preference will shift towards the overall norm.

37 participants were excluded from the analysis for either failing the understanding check (*n* = 23) and/or rating their attitude towards their chosen social issue as neutral (*n* = 14). The distribution of responses for the remaining 264 participants is shown in Fig 1.

#### Model comparison

In order to ascertain the extent to which self-categorization theory provides a better or worse explanation of our data, we directly compared these competing accounts using models that reflect their different assumptions, as described below.

Each of the following models represent Bayesian versions of ordinal logistic regression, which predicts the proportions of responses on an ordinal scale while assuming that certain variables (in our case, the descriptive norms) change the odds of making higher or lower responses on the scale. Specifically, the variables are parameterized in terms of the natural log odds of favouring a higher response (more strongly preferring to not report the robber). We can represent this as shown in Eq 1:

$${log}_{e}\left(\text{odds}\;\text{of}\;\text{responding}\;\text{higher}\right)=b_{in}I+b_{both}B+b_{out}I\times B$$ (1)

Here, *I* represents the ingroup descriptive norm condition and B represents the both norms shown condition. Because the option that was more popular with the ingroup was always less popular with the outgroup, we can represent both the presence and direction of the outgroup descriptive norm based on the interaction between the two independent variables (*I × B*). Meanwhile, *b*_*in*_, *b*_*both*_ *and b*_*out*_ *a*re parameters representing the effects of changing these conditions. The self-categorization and the alternative account make different assumptions about these parameters, which we represent using different priors, as outlined shortly.

One additional piece of data that the self-categorization model can make use of is whether the participants reported identifying with the ingroup and reported not identifying with the outgroup. According to self-categorization theory, individuals should only want to conform to a supposed ingroup norm if they actually self-identify with that group and should only want to avoid following an outgroup norm when they consider that outgroup as separate from their self-identity. We thus include these interactions in the self-categorization model, as outlined in Eq 2, where ingroup agree is coded as 1 if the participant reports identifying with the ingroup and 0 otherwise. Equivalent binary coding is used for outgroup disagree.

$${log}_{e}\left(\text{odds}\right)=b_{in}I\times \text{INGROUP}\;\text{AGREE}+b_{both}B+b_{out}I\times B\times \text{OUTGROUP}\;\text{DISAGREE}$$ (2)

The ingroup agree and outgroup disagree variables effectively act as switches, determining whether the self-categorization model assumes the participant will be affected by the ingroup and outgroup descriptive norms respectively. The alternative hypothesis assumes that identification with the group that a descriptive norm comes from does not influence the effect of that descriptive norm and thus, these variables are ignored by the alternative model. Out of the 264 participants included in this analysis, 219 (83%) identified with the ingroup and 220 (83%) did not identify with the outgroup. Scores on these two variables had no bearing on a participant’s inclusion in the data analysis, ensuring that the same participants are analysed for both the self-categorization and the alternative models.

#### Prior assumptions: b_in_

Given the similarity of our ingroup norm condition to the experiments reported in Pryor, Perfors and Howe [26], we used the observed effect of the ingroup descriptive norm in those experiments to inform our prior for the ingroup descriptive norm effect in the current analysis. The log odds ratio estimated across the relevant experiments from Pryor, Perfors and Howe [26] was 1.02 with a standard deviation of 0.19 (see S2 Text). A notable difference between those experiments and the descriptive norms that were presented in the current experiment is that the previous experiments presented a stronger ingroup descriptive norm (75% of ingroup did X) than the current experiment (60% of ingroup did X). To adjust for this difference and account for increased uncertainty in this parameter estimate, we set the prior distribution for the effect of the ingroup descriptive norm to be a folded normal distribution with a mean of $\frac{0.6}{0.75}\times 1.02=0.816$, and a standard deviation of 0.5 for both the self-categorization and alternative models. We folded this normal distribution such that the prior is restricted to be greater than 0, given both models assume that people’s preferences should shift towards the ingroup descriptive norm (i.e. the effect of the ingroup descriptive norm will be positive).

#### Prior assumptions: b_both_

The parameter *b*_*both*_ represents a possible main effect on responses of merely presenting both an ingroup and outgroup descriptive norm compared to only an ingroup norm, independent of the direction of those norms. Including this effect in the models is important as it allows for the possibility that the outgroup descriptive norm is more effective in one direction than in another. It also allows for the possibility that being presented with two opposing descriptive norms shifts people’s bias. For example, the increased ambiguity caused by having opposing norms presented may elicit an omission bias [27], wherein taking no action (i.e. not reporting the robber) is favoured more often, independent of the actual direction of the descriptive norms. Given that the presentation of the outgroup descriptive norm was independent of the direction of either norm, we had no clear, theoretical reason to predict a strong systematic effect in either direction due to merely presenting an outgroup descriptive norm, independent of that descriptive norm’s direction. Thus, the self-categorization and the alternative model both adopt a weakly informative prior for *b*_*both*_, represented by a normal distribution with a mean of 0 and standard deviation of 0.5.

#### Prior assumptions: b_out_

The parameter *b*_*out*_ is the key manner in which the self-categorization explanation of the descriptive norm effect differs from that of the alternative hypothesis that people conform to the overall descriptive norm. This parameter represents the extent to which presenting an outgroup descriptive norm that is opposite to the ingroup descriptive norm shifted preferences towards or away from the option favoured by the ingroup descriptive norm.

For self-categorization theory, presenting an outgroup descriptive norm that opposes the ingroup descriptive norm is expected to increase conformity with the ingroup norm. We represented this with a normally-distributed prior that was restricted to be greater than 0 (specifically a half-normal distribution with a mean of 0 and standard deviation of 0.5).

Contrasting with self-categorization theory, the alternative hypothesis assumes that people care about the overall descriptive norm, regardless of whether it comes from an ingroup or outgroup. Under it, ingroup and outgroup descriptive norms are assumed to affect preferences equivalently, only differing to the extent that the strength of these norms differ. Given that 85% of the outgroup was said to have favoured a particular option, compared to 60% of the ingroup favouring the alternative option, we represented this expectation by setting *b*_*out*_ to be a transformation of *b*_*in*_, such that $b_{out}={-\frac{0.85}{0.6}b}_{in}$.

#### Model comparison

We assessed the relative evidence for the self-categorization model and the alternative model provided by the data using a Bayes Factor (BF) calculated with the “Bridge Sampling” package in R [28]. This Bayes Factor represents the probability of the observed data occurring under the alternative model relative to the probability of the observed data occurring under the self-categorization model. Specifically,

$$BF=\frac{p(data|\text{alternative})}{p(data|\text{self-categorization})}$$

We found a BF of 34.97, suggesting that the observed data was 34.97 times more likely under the alternative model than under the self-categorization model. This represents strong evidence in favour of the alternative model over the self-categorization model. These results remain qualitatively the same across different values for the mean of the *b*_*out*_ prior (0, 0.5 and 1) and across differing values of the standard deviation of this prior (0.25, 0.5, 1 and 2), with the Bayes Factor always favoring the alternative model and ranging from 30.04 to 522.62.

**Fig 2:**
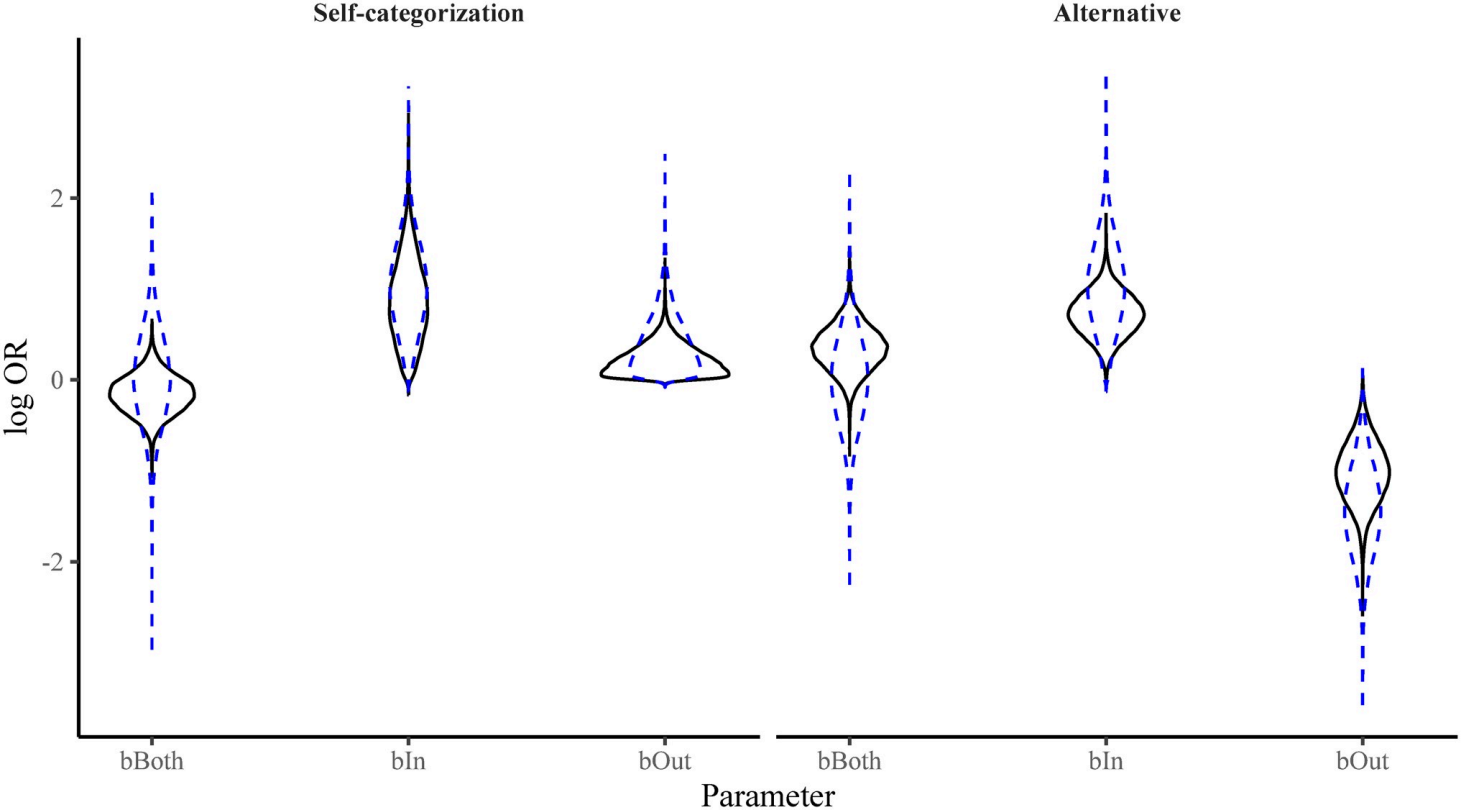
Violin plots of the prior (dashed blue lines) and posterior (solid black lines) density for each of the parameters in both the self-categorization model and the alternative model for Experiment 1. The results favour the alternative model over the self-categorization model.

Fig 2 shows the prior and posterior distribution of the parameters for each model. Even when we presented descriptive norms from ingroups and outgroups defined on issues that participants cared about and accounted for whether participants actually identified as ingroup members and did not identify with the outgroup (i.e. under conditions strongly favouring the self-categorization model), we did not find data consistent with self-categorization theory. Instead, these results support the alternative hypothesis that people tend to favour the overall descriptive norms, whether they identify with the people this norm relates to or not.

#### Effect sizes

As an additional analysis, we ran a frequentist ordinal logistic regression to measure the effect sizes for the parameters reported in Eq 1. We found a significant effect of the ingroup descriptive norm (*N* = 264, OR = 2.48, 95%CI[1.35, 4.58]), wherein participants’ preference shifted towards favouring the option that was popular under the ingroup descriptive norm. There was no significant shift in bias for participants who had both norms shown (*N* = 264, OR = 1.39, 95%CI[0.760, 2.55]). The observed interaction between ingroup descriptive norm and both norms shown (i.e. the effect of the outgroup descriptive norm) was in the direction consistent with the alternative hypothesis however, this effect was not significant (*N* = 264, OR = 0.429, 95%CI[0.181, 1.01]).

## Experiment 2

Experiment 1 found strong evidence against self-categorization theory, instead favouring the alternative hypothesis that people care more about the overall descriptive norm than about actively not conforming to outgroup descriptive norms. However, a frequentist analysis failed to reject the null hypothesis for the effect of the outgroup descriptive norm. Additionally, it may be the case that self-categorization mechanisms were not engaged due to the abstract nature of how we defined ingroups and outgroups. Self-categorization mechanisms may require a clearer social entity with which people can identify, such as political groups. We therefore conducted a pre-registered conceptual replication of Experiment 1, using political identity to define ingroups and outgroups, in order to ascertain whether the results from Experiment 1 were reliable. The pre-registration for this experiment is available at: https://osf.io/j9ugv.

### Method

#### Participants

600 English-speaking participants (M_age_ = 40 years, 47% female) from the United States of America agreed to participate via Mechanical Turk in exchange for US$0.65.

#### Procedure and design

Experiment 2 was exactly the same as Experiment 1 except that participants no longer selected and rated social issues that they cared about. Instead, participants were asked to rate their attitudes towards the two major US political parties (Republican and Democratic party) on an 11-point Likert scale ranging from “Strongly dislike” (-5) to “Strongly like” (+5). These ratings were used to define the ingroup and outgroup when subsequently presenting descriptive norms. Specifically, if the participant reported liking the Democratic party and disliking the Republican party, then the ingroup was Democratic party supporters while the outgroup was Republican party supporters and vice versa for those that reported liking the Republican party. For the 11 participants who reported disliking both the Republican and Democratic party, the ingroup was designated as Independents or other party supporters and their outgroup was designated as Republican or Democratic party supporters. This was reversed for the five participants that reported liking both parties. Twenty-five participants were excluded for rating both parties as neutral, preventing allocation of ingroups and outgroups.

### Results

**Fig 3:**
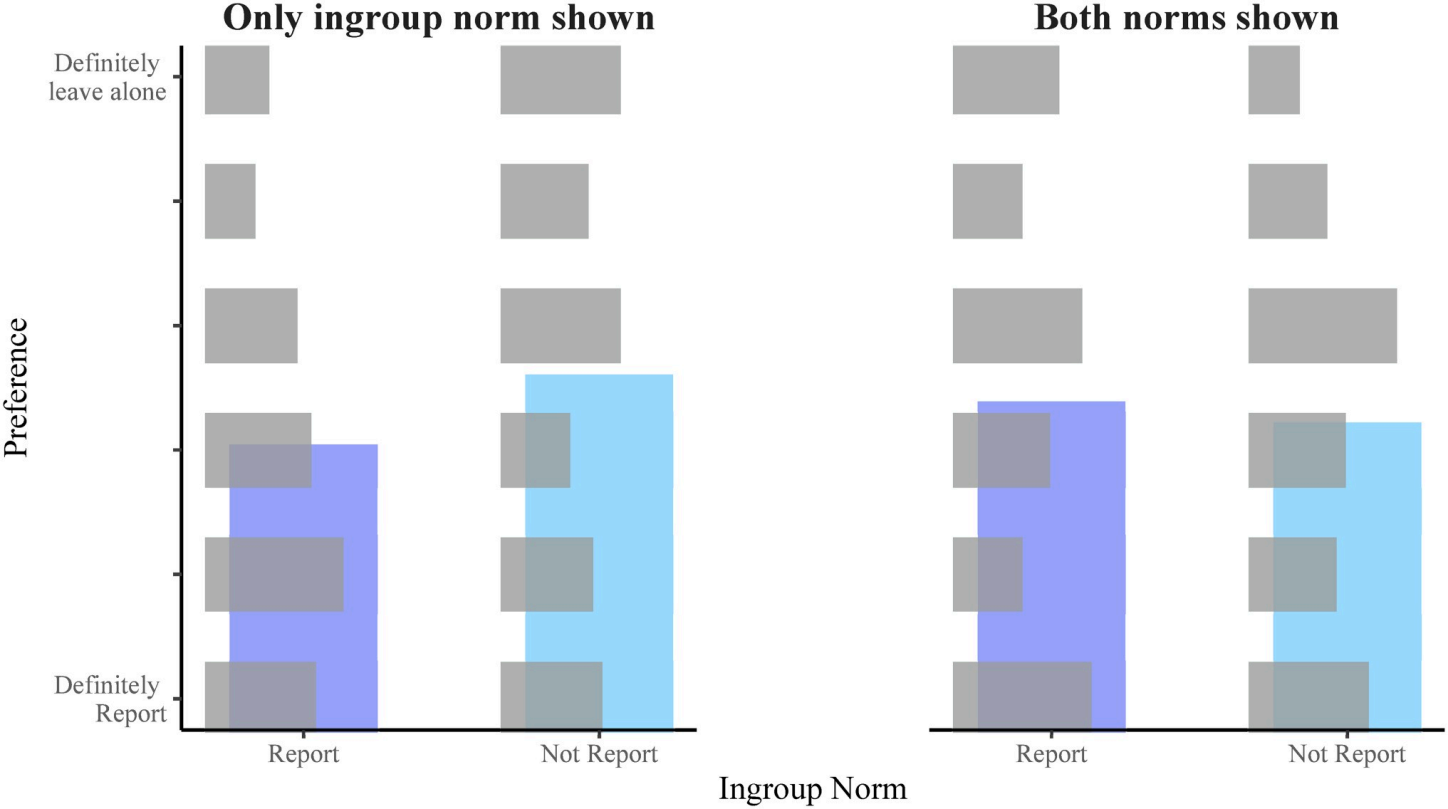
Distribution of responses by condition in Experiment 2. The horizontal grey bars represent the distribution of responses for each condition in Experiment 2. The vertical blue bars show the mean response for each condition to give an indication of the direction that responses shifted between conditions.

92 participants were excluded from the analysis for either failing the understanding check (*n* = 53) and/or rating both political parties as neutral (*n* = 42), preventing determination of an ingroup and outgroup. The distribution of responses for the remaining 508 participants is shown in Fig 3. Out of these participants, 428 (84%) identified with the ingroup and 437 (86%) did not identify with the outgroup, suggesting the allocation of ingroups and outgroups was generally, though not universally, successful.

An ordinal logistic regression found a significant effect of the ingroup descriptive norm (*N* = 508, OR = 1.79, 95%CI[1.16, 2.78]), wherein participants’ preferences tended to favour the ingroup descriptive norm. There was no significant shift in bias for participants who had both norms shown (*N* = 508, OR = 1.41, 95%CI[0.91, 2.18]). The observed interaction between ingroup descriptive norm and both norms shown (i.e. the effect of the outgroup descriptive norm), found that preferences significantly shifted towards the outgroup descriptive norm and away from the ingroup descriptive norm, consistent with the alternative hypothesis (*N* = 508, OR = 0.477, 95%CI[0.26, 0.88]).

**Fig 4:**
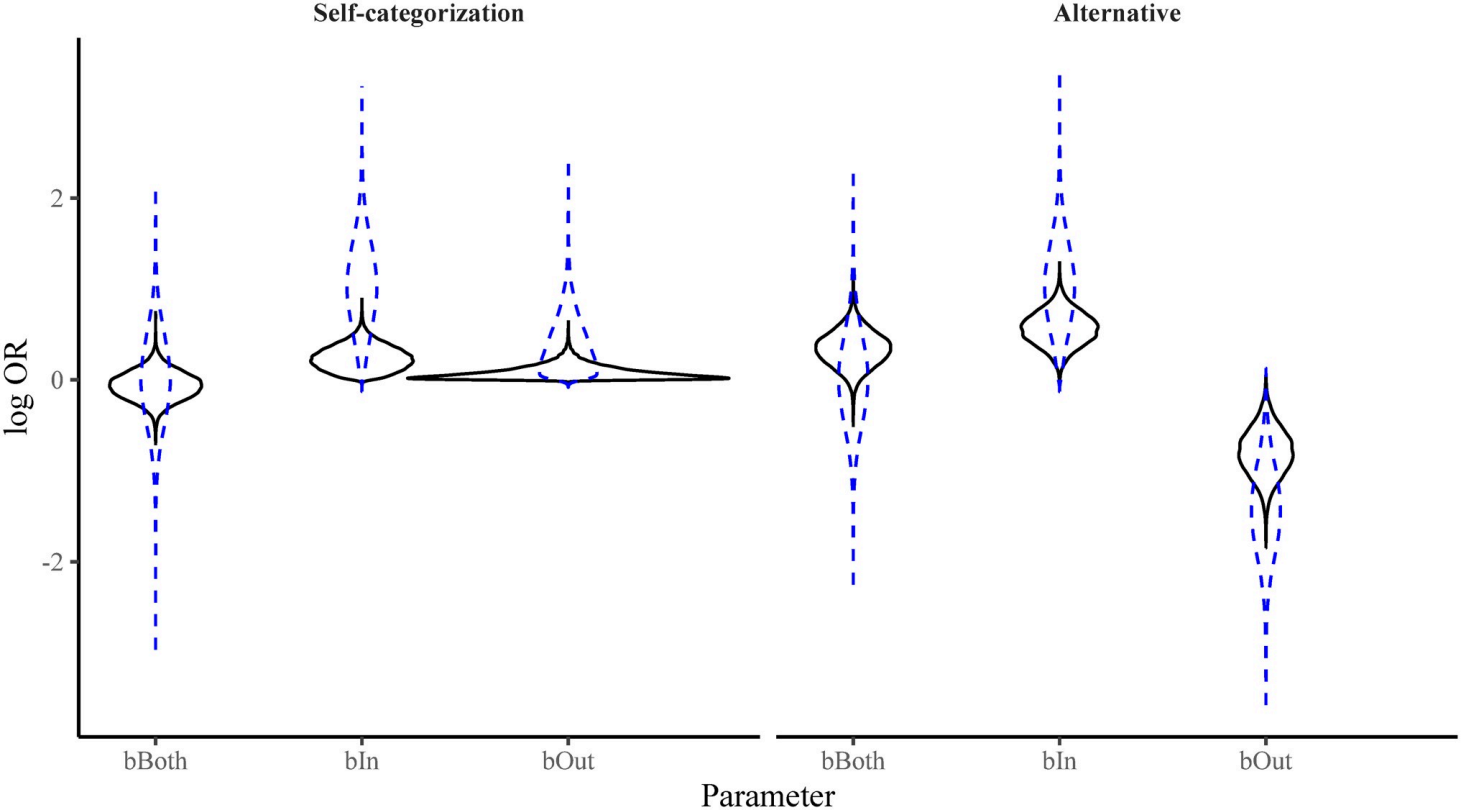
Violin plots of the prior (dashed blue lines) and posterior (solid black lines) density for each of the parameters in both the self-categorization model and the alternative model for Experiment 2. The results favour the alternative model over the self-categorization model.

Direct comparison of the self-categorization model and the alternative model found a BF of 286.31 in favour of the alternative model, showing strong evidence against the self-categorization model. Fig 4 shows the prior and posterior distribution of the parameters for each model. Even when we presented descriptive norms allegedly associated with political identity, participants’ responses tended to shift towards, rather than away from, the descriptive norm of the outgroup. If we collapse across both of the experiments reported in this paper, we find a Bayes Factor of 1272.50 in favour of the alternative model.

## Discussion

According to self-categorization theory, people are expected to actively conform to the norms of groups with which they identify at a given time (ingroups) and actively try to avoid conforming to the norms of groups with which they do not identify (outgroups) [9]. Across two experiments, we found evidence against this; participants shifted their preferences away from an option that was popular with an ingroup when they were additionally presented with an outgroup descriptive norm that favoured the alternative option. Interestingly, this was found even though ingroups and outgroups were defined based on political identity or social issues that participants themselves indicated that they personally cared strongly about (e.g. gun control). Thus, our findings provide strong evidence that self-categorization theory is unable to fully explain the descriptive norm effect. Instead, we suggest that people care more about following whichever behaviour is more popular overall.

It is worth emphasizing that our results do not prove that self-categorization never influences the descriptive norm effect. The fact we found results counter to self-categorization theory, even under conditions theoretically suited to it, suggests that self-categorization is less general than previously thought and cannot fully account for the descriptive norm effect. However, it may be that self-categorization contributes to the descriptive norm effect but only under more restricted conditions. For example, in the current paper, the descriptive norms were experimentally varied such that they had no pre-existing association with either the ingroup or outgroup. It may be that self-categorization only applies when a descriptive norm is strongly stereotypical of a group, such that it is a well-established part of ingroup or outgroup identity. Testing self-categorization theory under such conditions would be impractical though, given experimentally manipulating people’s ongoing stereotypes is difficult and likely unethical.

Alternatively, self-categorization mechanisms may be limited to when the descriptive norms are stronger. It may be that if a behaviour or preference is not essentially uniform across a group (i.e. approximately 100% of the group engages in it) then it is not considered a salient part of that group’s identity. In the current paper, we used descriptive norms that were notably lower than 100%. Perhaps presenting norms that are closer to 100% might be considered more relevant to group identity and thus, self-categorization mechanisms might be more engaged. Given most descriptive norms, both appearing in research and the real-world, are generally weaker than 100%, this would substantially limit the relevance of self-categorization theory. Nevertheless, the extent to which varying the strength of descriptive norms changes the mechanisms driving their influence would be a compelling area for future research.

We are also not arguing that people do not distinguish between the ingroup and the outgroup. The alternative model specified in this paper made a simplifying assumption that, ingroup and outgroup descriptive norms would have equivalent effects on people’s preference assuming they are of equal strength. This is an unrealistic assumption and likely served to hinder the alternative model. Pryor, Perfors and Howe [26], for example, presented norms from an ingroup and outgroup of equal strength and found that participants tended to conform more to the ingroup norm than the outgroup norm. While people’s responses to ingroup and outgroup norms may tend to differ in degree, our results suggest that they do not differ in direction.

In terms of the generality of this finding, it is worth noting that we focused exclusively on descriptive norms, relating to what most other people do. Another important social pressure involves how most other people feel we ought to behave, termed injunctive norms. Descriptive and injunctive norms do not always align [29] and can have distinct effects [30]. Given injunctive norms more directly relate to the attitudes of other people, it is possible that they are more relevant to group identity and thus, may be more likely to engage self-categorization mechanisms. Nevertheless, it has been found that people tend to automatically and implicitly infer the existence of injunctive norms when informed about descriptive norms [31], so it is possible our participants were not only influenced by our explicitly presented descriptive norms but also by their inferred injunctive norms.

Our data shows that, at least in some circumstances, people tend to conform to descriptive norms even when they are exhibited by an outgroup. There are a number of reasons why this might occur. One possible reason is that people may have an evolved or conditioned anxiety response to deviating from the overall descriptive norm. If everyone else is engaging in a behaviour, it is often because that behaviour has been deemed beneficial by others [32] and deviating from it may cause you to stand out and expose yourself to additional risk [33]. Psychological studies [34, 35], sociological studies [36] and agent-based simulations [37] have shown that conformity or societal norms tend to be stronger when the society or individuals within it are threatened. Thus, people may conform to norms simply because they feel anxious otherwise.

Another possible reason why individuals may conform to the overall descriptive norm, regardless of group status, might be to diffuse responsibility. Studies have shown people experience more regret for bad outcomes that result from actions rather than simply omitting to act [38–40]. In the case of descriptive norms, it may be that people can offload the sense of responsibility for a decision if most other people are engaging in it. Acting differently to how most people are behaving requires taking on a greater level of personal responsibility for the decision, increasing the potential for anticipated regret. Consistent with the idea that anticipated regret is reduced when offloading the responsibility to others, Steffel and Williams [41] found that regret decreased when delegating a decision to someone else, even when the outcome of the decision was the same and that delegate was inexperienced. Similarly, this diffusion of responsibility may also decrease the level of guilt people feel when following a descriptive norm, as seen in the bystander effect [42].

One final reason why people may tend to conform to the overall norm is that they view it as the status quo. Studies have found that people like to maintain the current situation rather than enact change [43, 44]. A similar reason may lead people to conform with norms. The most prominent explanation for these status quo biases is loss aversion [45]. If people treat the overall descriptive norm as a reference point, then they may tend to evaluate all other options relative to it. Any way in which an option is better than the overall descriptive norm will be seen as a gain while any way in which an option is worse than the overall descriptive norm will be seen as a loss. The notion of loss aversion then suggests that these losses loom larger than gains, such that shifting away from the overall descriptive norm tends to have a net negative value. However, the moral dilemmas presented in the current paper were hypothetical and, even hypothetically, did not offer any clear personal gains or losses to the participant. Thus, unless the notion of loss aversion extends beyond personal outcomes, it may not be able to explain our particular findings.

The current study was not designed to distinguish between these competing reasons as to why people may tend to adhere to the overall descriptive norm. Further work would be needed to determine which processes are important in which circumstances. For example, if people follow the overall descriptive norm because it allows them to reduce their sense of guilt in the event that their action has negative consequences, we would expect the descriptive norm effect to be stronger when the decision has important consequences external to the participant, increasing the potential level of guilt. In contrast, loss aversion and anticipated regret explanations would predict the effect to be stronger when the decision has large personal consequences for the participant. If people follow norms due to a conditioned or evolved anxiety response to violating norms, then they should be influenced by the descriptive norm just as much, regardless of the above changes, provided anxiety is controlled for. All of these alternative explanations we have offered suggest that negative emotions such as guilt and anxiety are increased when deviating from a norm. Self-report and physiological tests could look at whether this is the case or whether entirely different explanations are needed.

Though this paper is theoretically driven, the fact that descriptive norms are commonly used in real-world behavioural interventions encourages consideration of the real-world implications of our findings. The obvious implication of our results is that people may tend to conform to popular behaviour, even when that behaviour is predominantly engaged in by outgroup members. For example, if a particular social group tends to engage in an undesirable, polarized behaviour, our findings suggest that increasing exposure of these individuals to people outside their group is likely to decrease the incidence of their undesirable behaviour. In contrast, self-categorization theory predicts that increased exposure to outgroups would encourage such individuals to further polarize towards their socially undesirable behaviour.

In this paper we found that people’s preference shifted towards an option that was popular based on descriptive norms, even when the option was popular amongst outgroup members. This occurred even when the outgroup was based on strongly divisive issues such as political affiliation or important social issues. Prior to this research, it would have been intuitive to predict that people would actively avoid following the norms of outgroups they are clearly opposed to (such as an opposing political party). This prediction is made by self-categorization theory, a particularly prominent explanation of the descriptive norm effect. Thus, self-categorization theory cannot explain our results. Instead, other theoretical explanations of the descriptive norm effect are needed that assume people have a more general desire to conform towards norms, even when they come from an outgroup. We have considered how a number of more general mechanisms drawn from broader findings on conditioned emotional responses, anticipated regret and reference point effects could potentially explain our findings.

## Supporting information

S1 Text Example transcript. (DOCX)

S2 Text Description of prior analysis. (DOCX)

S3 Text Description of issue statements. (DOCX)

## References

[1] Asch SE. Effects of group pressure upon the modification and distortion of judgments In: Guetzkow H, editor. Groups, Leadership, and Men. Pittsburgh, PA: Carnegie Press; 1951 p. 177–90.

[2] Sherif M. The psychology of social norms. New York & London: Harper & Brothers Publishing; 1936.

[3] Wenzel M. Misperceptions of social norms about tax compliance: From theory to intervention. Journal of Economic Psychology. 2005;26(6): 862–83.

[4] Schultz PW, Nolan JM, Cialdini RB, Goldstein NJ, Griskevicius V. The constructive, destructive, and reconstructive power of social norms. Psychological science. 2007;18(5): 429–34. 10.1111/j.1467-9280.2007.01917.x 17576283

[5] Cabinet Office, The Behavioural Insights Team, Department of Health, Driver and Vehicle Licensing Agency, NHS Blood and Transplant. Applying behavioural insights to organ donation: Preliminary results from a randomised controlled trial. London: Cabinet Office; 2013.

[6] Abbink K, Freidin E, Gangadharan L, Moro R. The effect of social norms on bribe offers. The Journal of Law, Economics, and Organization. 2018;34(3): 457–74.

[7] Köbis NC, Van Prooijen J-W, Righetti F, Van Lange PA. “Who doesn’t?”—The impact of descriptive norms on corruption. PloS one. 2015;10(6): e0131830 10.1371/journal.pone.0131830 26121127 PMC4487686

[8] Bicchieri C, Xiao E. Do the right thing: but only if others do so. Journal of Behavioral Decision Making. 2009;22(2): 191–208.

[9] Hogg MA, Turner JC, Davidson B. Polarized Norms and Social Frames of Reference: A Test of the Self-Categorization Theory of Group Polarization. Basic and Applied Social Psychology. 1990;11(1): 77–100.

[10] Tajfel H. Experiments in intergroup discrimination. Scientific American. 1970;223(5): 96–103. 5482577

[11] Haslam SA, Oakes PJ, McGarty C, Turner JC, Reynolds KJ, Eggins RA. Stereotyping and social influence: The mediation of stereotype applicability and sharedness by the views of in-group and out-group members. British Journal of Social Psychology. 1996;35(3): 369–97.

[12] Turner JC. Explaining the nature of power: A three-process theory. European journal of social psychology. 2005;35(1): 1–22.

[13] Turner JC, Hogg MA, Oakes PJ, Reicher SD, Wetherell MS. Rediscovering the social group: A self-categorization theory. Oxford, UK: Basil Blackwell; 1987.

[14] Wellen JM, Hogg MA, Terry DJ. Group norms and attitude–behavior consistency: The role of group salience and mood. Group Dynamics: Theory, Research, and Practice. 1998;2(1): 48–56.

[15] Smith JR, Terry DJ. Attitude-behaviour consistency: The role of group norms, attitude accessibility, and mode of behavioural decision-making. European Journal of Social Psychology. 2003;33(5): 591–608.

[16] Rimal RN. Modeling the relationship between descriptive norms and behaviors: A test and extension of the theory of normative social behavior (TNSB). Health Communication. 2008;23(2): 103–16. 10.1080/10410230801967791 18443998

[17] Rimal RN, Real K. How behaviors are influenced by perceived norms: A test of the theory of normative social behavior. Communication Research. 2005;32(3): 389–414.

[18] Rimal RN, Real K. Understanding the influence of perceived norms on behaviors. Communication Theory. 2003;13(2): 184–203.

[19] Rimal RN, Lapinski MK, Cook RJ, Real K. Moving toward a theory of normative influences: How perceived benefits and similarity moderate the impact of descriptive norms on behaviors. Journal of Health Communication. 2005;10(5): 433–50. 10.1080/10810730591009880 16199387

[20] Tajfel H, Billig MG, Bundy RP, Flament C. Social categorization and intergroup behaviour. European journal of social psychology. 1971;1(2): 149–78.

[21] Krizan Z, Baron RS. Group polarization and choice-dilemmas: How important is self-categorization? European Journal of Social Psychology. 2007;37(1): 191–201.

[22] Cruwys T, Platow MJ, Angullia SA, Chang JM, Diler SE, Kirchner JL, et al Modeling of food intake is moderated by salient psychological group membership. Appetite. 2012;58(2): 754–7. 10.1016/j.appet.2011.12.002 22178007

[23] Greene S. Understanding Party Identification: A Social Identity Approach. Political Psychology. 1999;20(2): 393–403.

[24] Kittur A, Chi EH, Suh B, editors. Crowdsourcing user studies with Mechanical Turk. Proceedings of the SIGCHI conference on human factors in computing systems; 2008: ACM.

[25] Postmes T, Haslam SA, Jans L. A single-item measure of social identification: Reliability, validity, and utility. British journal of social psychology. 2013;52(4): 597–617. 10.1111/bjso.12006 23121468

[26] Pryor CG, Perfors A, Howe PDL. Even arbitrary norms influence moral decision-making. Nature Human Behaviour. 2019;3: 57–62. 10.1038/s41562-018-0489-y 30932055

[27] Ritov I, Baron J. Reluctance to vaccinate: Omission bias and ambiguity. Journal of Behavioral Decision Making. 1990;3(4): 263–77.

[28] Gronau QF, Sarafoglou A, Matzke D, Ly A, Boehm U, Marsman M, et al A tutorial on bridge sampling. Journal of mathematical psychology. 2017;81: 80–97. 10.1016/j.jmp.2017.09.005 29200501 PMC5699790

[29] Park HS, Smith SW. Distinctiveness and Influence of Subjective Norms, Personal Descriptive and Injunctive Norms, and Societal Descriptive and Injunctive Norms on Behavioral Intent: A Case of Two Behaviors Critical to Organ Donation. Human Communication Research. 2007;33(2): 194–218. 10.1111/j.1468-2958.2007.00296.x

[30] Melnyk V, Herpen Ev, Fischer AR, van Trijp H. To think or not to think: the effect of cognitive deliberation on the influence of injunctive versus descriptive social norms. Psychology & marketing. 2011;28(7): 709–29.

[31] Eriksson K, Strimling P, Coultas JC. Bidirectional associations between descriptive and injunctive norms. Organizational Behavior and Human Decision Processes. 2015;129: 59–69.

[32] Cialdini RB, Reno RR, Kallgren CA. A focus theory of normative conduct: Recycling the concept of norms to reduce littering in public places. Journal of Personality and Social Psychology. 1990;58(6): 1015–26.

[33] Hamilton WD. Geometry for the selfish herd. Journal of theoretical Biology. 1971;31(2): 295–311. 5104951 10.1016/0022-5193(71)90189-5

[34] Sarnoff I, Zimbardo PG. Anxiety, fear, and social isolation. The Journal of Abnormal and Social Psychology. 1961;62(2): 356–63. 13746557 10.1037/h0046506

[35] Darley JM. Fear and social comparison as determinants of conformity behavior. Journal of personality and social psychology. 1966;4(1): 73–8. 5966183 10.1037/h0023508

[36] Gelfand MJ, Raver JL, Nishii L, Leslie LM, Lun J, Lim BC, et al Differences between tight and loose cultures: A 33-nation study. science. 2011;332(6033): 1100–4. 10.1126/science.1197754 21617077

[37] Roos P, Gelfand M, Nau D, Lun J. Societal threat and cultural variation in the strength of social norms: An evolutionary basis. Organizational Behavior and Human Decision Processes. 2015;129: 14–23.

[38] Nicolle A, Fleming SM, Bach DR, Driver J, Dolan RJ. A regret-induced status quo bias. The journal of Neuroscience. 2011;31(9): 3320–7. 10.1523/JNEUROSCI.5615-10.2011 21368043 PMC3059787

[39] Baron J, Ritov I. Reference Points and Omission Bias. Organizational Behavior and Human Decision Processes. 1994;59(3): 475–98. 10.1006/obhd.1999.2839 10433898

[40] Kahneman D, Miller DT. Norm theory: Comparing reality to its alternatives. Psychological review. 1986;93(2): 136–53.

[41] Steffel M, Williams EF. Delegating Decisions: Recruiting Others to Make Choices We Might Regret. Journal of Consumer Research. 2018;44(5): 1015–32.

[42] Darley JM, Latane B. Bystander intervention in emergencies: Diffusion of responsibility. Journal of personality and social psychology. 1968;8(4): 377–83. 5645600 10.1037/h0025589

[43] Thaler R. Toward a positive theory of consumer choice. Journal of Economic Behavior & Organization. 1980;1(1): 39–60.

[44] Samuelson W, Zeckhauser R. Status quo bias in decision making. Journal of risk and uncertainty. 1988;1(1): 7–59.

[45] Kahneman D, Tversky A. Prospect theory: An analysis of decision under risk. Econometrica. 1979;47(2): 263–92.

