# Supplementary material for: Conformity to the descriptive norms of people with opposing political or social beliefs
Source: PLoS One. 2019 Jul 10;14(7):e0219464. doi: 10.1371/journal.pone.0219464 (PMC6619767; doi:10.1371/journal.pone.0219464)
Supplement: S1 Text — (DOCX) [file pone.0219464.s001.docx]

# S1 Example Transcript

The following is an example transcript of the instructions in Experiment 1 that could have been presented to a participant that indicated they cared strongly about gun control.

## Instructions

*We are following up on a previously published paper that looked at how people feel about moral dilemmas.*

*In the previous paper, a moral dilemma was described that involved two possible courses of actions. Participants chose which action they preferred and had to rate how they would feel about performing that action.*

*In this study, you will be presented with a scenario describing a moral dilemma. You will choose which action you would take and then provide a rating of how good or bad you imagine you would feel after taking that action.*

## Experimental trial

*Imagine you have witnessed a man rob a bank. However, you then saw him do something unexpected with the money. He donated it all to a run-down orphanage that would benefit greatly from the money. You must decide whether to call the police and report the robber or do nothing and leave the robber alone.*

In the previous study:

- approximately 60% of participants who agreed with you about gun restrictions chose to call the police and report the robber.
- approximately 85% of participants who disagreed with you about gun restrictions chose to do nothing and leave the robber alone.

Would you:

- *Definitely call the police and report the robber*
- *Very likely call the police and report the robber*
- *Probably call the police and report the robber*
- *Probably do nothing and leave the robber alone*
- *Very likely do nothing and leave the robber alone*
- *Definitely do nothing and leave the robber alone*

## Rating choice

*You chose to call the police and report the robber. If you did call the police and report the robber, how would you expect to feel:*

- *Very good*
- *Moderately good*
- *Slightly good*
- *Neither good or bad*
- *Slightly bad*
- *Moderately bad*
- *Very bad*

## Understanding check

*We were following up on a previous study in this task. Given what we described in the instructions, which of the following is true about the previous study?*

- *Participants chose which action they preferred* (correct)
- *Due to a computer error, participants were not allocated equally to imagine performing the different actions* (incorrect)
- *No data was saved during the experiment.* (incorrect)
- *The participants completed the experiment with their eyes closed.* (incorrect)

## Identity Check

*Please rate how much you agree or disagree with the following statements:*

- *I identify with Pro-Gun Enthusiasts*
- *I identify with Anti-Gun Advocates*
