# Supplementary material for: Conformity to the descriptive norms of people with opposing political or social beliefs
Source: PLoS One. 2019 Jul 10;14(7):e0219464. doi: 10.1371/journal.pone.0219464 (PMC6619767; doi:10.1371/journal.pone.0219464)
Supplement: S2 Text — (DOCX) [file pone.0219464.s002.docx]

# S2 Prior Analysis

We ran a Bayesian ordinal logistic regression on the data across Experiments 1A, 1B and 2 from Pryor, Perfors and Howe (2019) to inform the priors for the current study. The data from those experiments is available at the following repository: https://osf.io/n6uz5/?view_only=7dc67fcc0c1f4fdea8fe1dfe5d492480.

We predicted responses to the moral dilemma as a function of whether the ingroup norm favored reporting the robber or leaving the robber alone. We placed a weakly regularizing prior on the effect of the ingroup norm (measured as a log odds ratio) using a normal distribution with a mean of 0 and a SD of 10. The posterior distribution for the effect of the ingroup norm had a mean of 1.27 and a SD of 0.24, and is shown in Supplementary Fig S2. We used this posterior to inform some of the priors for the current paper.


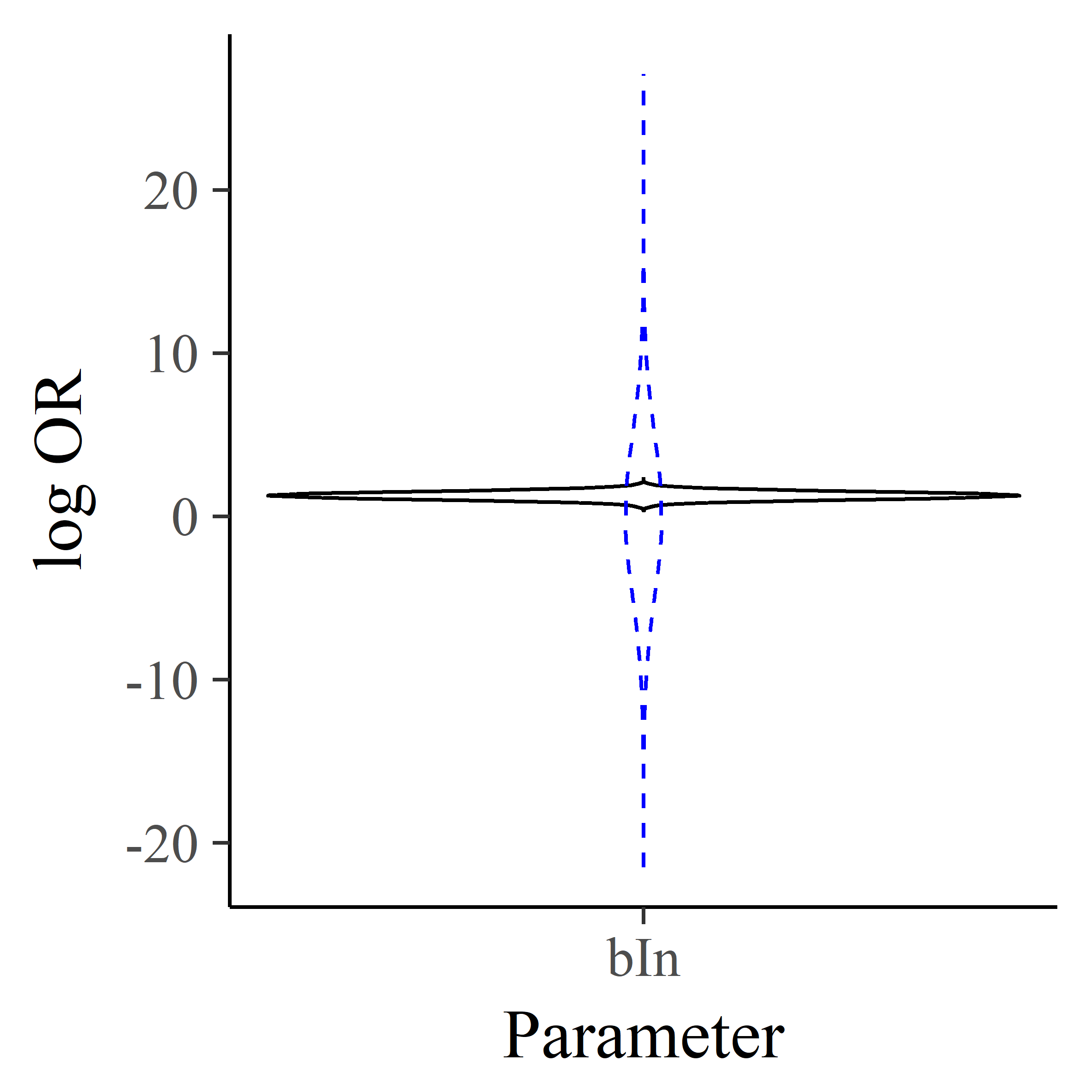


***Fig S2.* Violin plot of the prior (dashed blue line) and posterior (solid black line) density for the effect of the ingroup norm in Pryor, Perfors & Howe (2019).**
