# Supplementary material for: Conformity to the descriptive norms of people with opposing political or social beliefs
Source: PLoS One. 2019 Jul 10;14(7):e0219464. doi: 10.1371/journal.pone.0219464 (PMC6619767; doi:10.1371/journal.pone.0219464)
Supplement: S3 Text — (DOCX) [file pone.0219464.s003.docx]

# S3 Issue Statements

Following is the list of issues and corresponding statements that participants were presented with in Experiment 1. Supplementary Fig S3 shows the joint frequency with which participant chose each issue as the one they cared about most, and the extent to which they then agreed or disagreed with the subsequent statement about that issue.

- **Gun control:** “Adults should have the right to carry a concealed handgun”
- **Feminism:** “Feminism is important and beneficial to modern society”
- **Donald Trump:** “Donald Trump being president is good for the United States at this time”
- **Immigration and Dreamers:** "Dreamers (undocumented immigrants who came to the US as children) should be allowed to stay in the United States"
- **Transgender rights:** “Transgender people should be allowed to use the bathrooms of the gender they identify as"
- **Drug legalization:** "Possession of drugs should be legalized"
- **Colin Kaepernick kneeling during the national anthem:** “"Colin Kaepernick was wrong to kneel during the national anthem"
- **Buying and wearing fur:** "Buying and wearing fur is wrong"
- **Taxing religious organization:** "Religious organizations should be taxed"


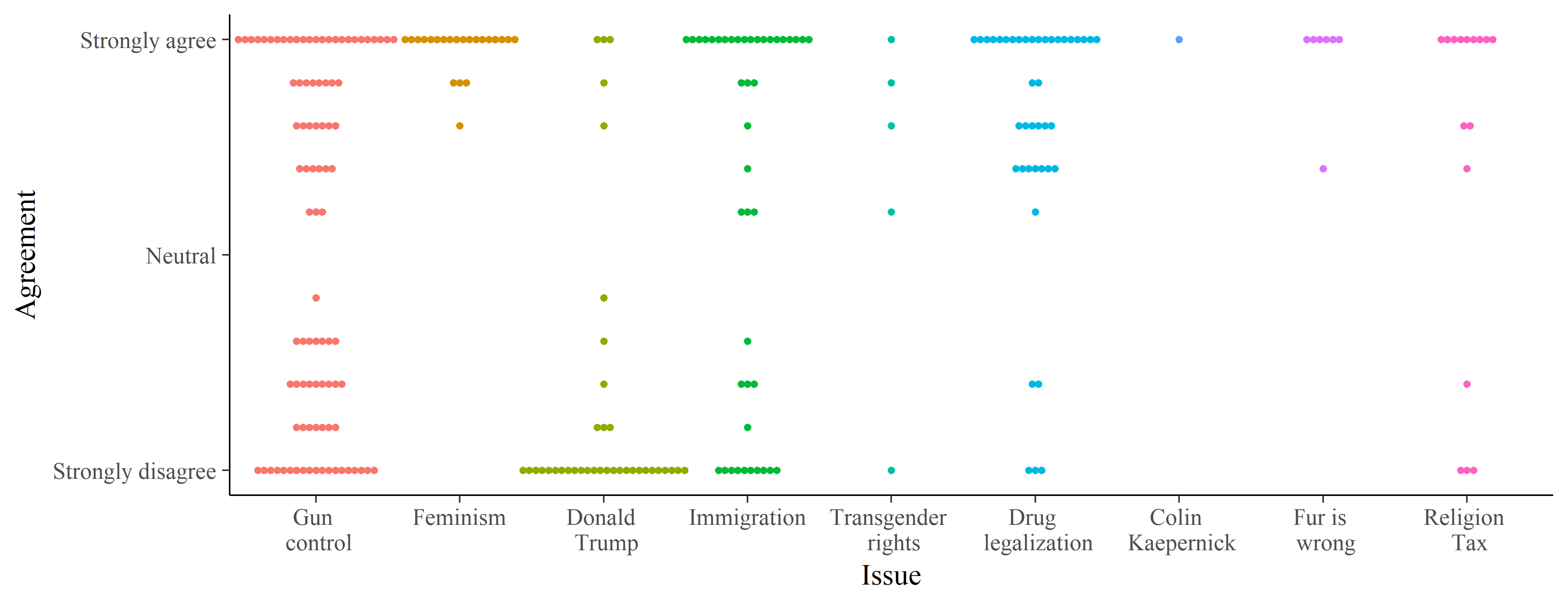
 ***Fig S3*. Dot plot of the joint frequency of participants caring most about an issue and the extent to which they then agreed with the statement about that issue in Experiment 1.**
